# Purification of phage display-modified bacteriophage T4 by affinity chromatography

**DOI:** 10.1186/1472-6750-11-59

**Published:** 2011-05-31

**Authors:** Anna Oślizło, Paulina Miernikiewicz, Agnieszka Piotrowicz, Barbara Owczarek, Agnieszka Kopciuch, Grzegorz Figura, Krystyna Dąbrowska

**Affiliations:** 1 Bacteriophage Laboratory, Institute of Immunology and Experimental Therapy, Polish Academy of Sciences, R. Weigla 12, Wroclaw, 53-114, Poland

## Abstract

**Background:**

Affinity chromatography is one of the most efficient protein purification strategies. This technique comprises a one-step procedure with a purification level in the order of several thousand-fold, adaptable for various proteins, differentiated in their size, shape, charge, and other properties. The aim of this work was to verify the possibility of applying affinity chromatography in bacteriophage purification, with the perspective of therapeutic purposes. T4 is a large, icosahedral phage that may serve as an efficient display platform for foreign peptides or proteins. Here we propose a new method of T4 phage purification by affinity chromatography after its modification with affinity tags (GST and Histag) by *in vivo *phage display. As any permanent introduction of extraneous DNA into a phage genome is strongly unfavourable for medical purposes, integration of foreign motifs with the phage genome was not applied. The phage was propagated in bacteria expressing fusions of the phage protein Hoc with affinity tags from bacterial plasmids, independently from the phage expression system.

**Results:**

Elution profiles of phages modified with the specific affinity motifs (compared to non-specific phages) document their binding to the affinity resins and effective elution with standard competitive agents. Non-specific binding was also observed, but was 10^2^-10^5 ^times weaker than the specific one. GST-modified bacteriophages were also effectively released from glutathione Sepharose by proteolytic cleavage. The possibility of proteolytic release was designed at the stage of expression vector construction. Decrease in LPS content in phage preparations was dependent on the washing intensity; intensive washing resulted in preparations of 11-40 EU/ml.

**Conclusions:**

Affinity tags can be successfully incorporated into the T4 phage capsid by the *in vivo *phage display technique and they strongly elevate bacteriophage affinity to a specific resin. Affinity chromatography can be considered as a new phage purification method, appropriate for further investigations and development.

## Background

Phage particle purification is important for two different issues: general investigation of bacteriophage particles, i.e. phage biology studies, and for therapeutic applications of bacteriophages. The first issue successfully applies gradient centrifugation of bacteriophage lysates, in caesium or saccharose [1-3]. In this case the limiting factor is mainly the amount of a bacteriophage batch that can be obtained by a single round of centrifugation. Nevertheless, the method can be sufficient for many laboratory-scale applications. Therapeutic use of bacteriophages requires large-scale preparations that may be obtained by various chromatography techniques [4-6]. In these techniques bacteriophages are generally expected to behave as "protein-like" fractions with no specificity. This approach probably provides the best results, although most bacteriophages are spatially expanded polyhedrons with very long tails, different from single protein molecules. Bacteriophages also constitute a very diverse and non-homogeneous group [7]. Therefore any methods are effective usually only for a selected group of phage strains. The problem of effective removal of protein and non-protein (mainly lipopolysaccharide: LPS) bacterial residuals still limits the therapeutic applications of some phages. So that the meaning is clear in acute infections, patients of a poor general condition, low immunological status, and in cases that apparently require parenteral injections. Even investigations of phage impact on higher organisms, i.e. immunological and other physiological assays *in vivo*, often require large amounts of highly purified phages. In these cases currently used procedures still do not provide satisfactory results and there is an important need to develop phage purification methods.

Affinity chromatography is one of the most efficient protein purification strategies [8]. This technique comprises a one-step procedure with a purification level in the order of several thousand-fold, adaptable for various proteins, heterogeneous in their size, shape, charge, and other properties. Affinity chromatography is based on interactions of an affinity tag, genetically incorporated into the protein of interest, and a carbohydrate resin, which is enriched with a specific, tag-binding motif/agent. After expression in bacteria (or other), the recombined target protein is able to interact specifically with the resin. Therefore washing of all other proteins and contaminations, and elution of the protein are possible. Moreover, this is usually simple and effective. Introducing affinity chromatography into the methods of bacteriophage purification can result in a simple and effective procedure, but it requires the placement of specific affinity tags on bacteriophage capsids.

The technique that enables fusion of a foreign peptide, protein domain or even a relatively large protein with a structural protein of a viral particle is phage display. Foreign peptides are presented on the outer surface of a viral coat, often in many copies per capsid [9]. It is not complicated to introduce short oligopeptides, and filamentous phages have been extensively used in these kinds of modifications. Icosahedral phages, e.g. lambda phage or T7, can serve as efficient platforms for large protein display [10-12].

T4 is also one of the large, icosahedral phages that may serve as a display platform. Importantly, it is not lysogenic (in contrast to the lambda phage), which has often been postulated as a requisite of therapeutic phages [13]. It also represents a numerous phage group (T4-like phages) sharing substantial homologies and similarities, and its genome and proteome are very well described [14,15]. Therefore T4 is a potent model for general investigations. The T4 bacteriophage capsid has been modified successfully with extra protein motifs several times. Fully active anti-lysozyme IgG, two domains of the HIV1-CD4 receptor, and PorA peptide from *Neisseria meningitidis *were fused to expose capsid proteins Soc and Hoc and displayed on the T4 capsid surface [16-18]. These modifications of the phage were achieved with the "*in vivo *phage display technique", i.e. natural assemblage in bacteria during a lytic growth cycle was employed for introducing fusion proteins to the phage capsid. The fusion comprised gpSoc or gpHoc and the protein/peptide of interest. The host bacteria expressed the fusion proteins from a designed expression vector or fusion protein was generated by integration of tag-coding sequences to the phage genome. The T4 phage strains used in the experiments with supplementary expression vectors had a deletion of *soc *or a non-sense mutation in the *hoc *gene, and thus no native gene products (Hoc or Soc) were incorporated into its head during phage assembly. Since Hoc and Soc are not essential head proteins, these defects do not affect phage viability [16-18].

Bacteriophage T4 was also found applicable for multi-component anthrax toxin and for HIV antigen presentation in *in vitro *phage display (prepared capsids of T4 *hoc*^- ^*soc*^- ^were completed *in vitro *with purified recombinant Hoc) [19-21].

Here we propose a new method of T4 phage purification by affinity chromatography after its modification with affinity tags (GST and Histag) by *in vivo *phage display. This work was based on previous observations of T4 phage capsid display capacity by Ren and Black [16] that were combined with standard experience in recombinant protein purification by affinity chromatography. As any permanent introduction of extraneous DNA into a phage genome is strongly unfavourable for therapeutic purposes, integration of foreign motifs with the phage genome was not applied. The phage was propagated in bacteria expressing fusions of its proteins with affinity tags.

## Results

**Figure 1:**
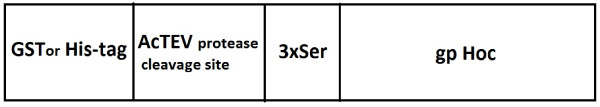
**Schema of the expression product used for supplementation of gpHoc defect in the HAP1 phage: fusion of gpHoc, 3-serine highly soluble motive (for effective proteolysis), sequence recognised by AcTEV protease, and the affinity tag (GST or 6-histidine motive)**.

**Figure 2:**
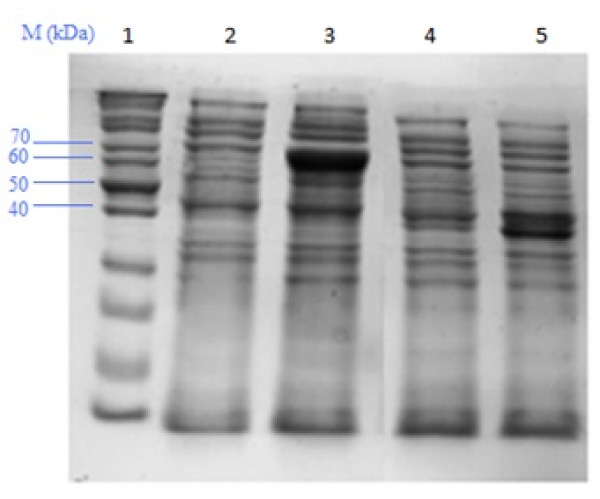
**Expression of gpHoc-GST and gpHoc-His tag**. Marker (Fermentas SM0661) Total protein profile in the non-induced *E. coli *strain transformed with the expression pDEST15 + *hoc *vector Total protein profile in the IPTG-induced *E. coli *strain transformed with the expression pDEST15 + *hoc *vector: overexpression of Hoc-GST (approx. 70 kDa) Total protein profile in the non-induced *E. coli *strain transformed with the expression pDEST17+*hoc *vector Total protein profile in the IPTG-induced *E. coli *strain transformed with the expression pDEST17 + *hoc *vector: overexpression of Hoc-GST (approx. 40 kDa)

Expression of the fusion proteins gpHoc with affinity tags (GST or His tag) was tested in an expression *E. coli *strain before the procedure of phage capsid modification by phage display. Effective production of the recombined proteins was observed both for the vector coding GST and the vector coding His tag (Figure 1 and 2).

HAP1 phage was applied as the platform for the display; this phage is defective in the gene *hoc*, i.e. gpHoc is not incorporated into the phage capsid [22]. HAP1 takes the place of other Hoc-deprived T4 strains described in previous studies on Hoc-based phage display by Ren and Black [16], and by Shivachandra et al. [20,21]. It is not a specific strain for this work and can be replaced with another strain derived from T4 but lacking gpHoc. The expression vectors were used for simultaneous expression of fusion proteins and propagation of bacteriophage HAP1 in *E. coli*, i.e. phage display *in vivo*. In this procedure the phage was expected to incorporate into its capsid gpHoc combined with affinity tags. Lysis of bacterial expressive cells was observed and the phage titre was determined in the clarified and filtered lysates.

**Figure 3:**
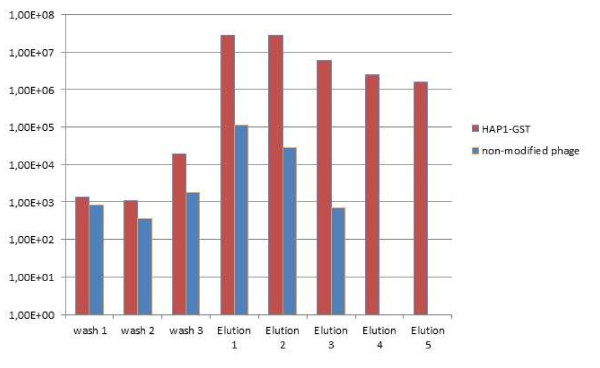
**Elution profile for the bacteriophage HAP1 modified with GST tag and purified on glutathione Sepharose compared to a non-modified phage**. wash 1 - phage concentration in the washing flow-through (1^st ^litre of washing buffer) wash 2 - phage concentration in the washing flow-through (2^nd ^litre of washing buffer) wash 3 - phage concentration in the final washing fraction (3^rd ^litre of washing buffer) elution 1 - phage concentration in the first elution fraction elution 2 - phage concentration in the second elution fraction elution 3 - phage concentration in the third elution fraction elution 4 - phage concentration in the fourth elution fraction (modified phage only) elution 5 - phage concentration in the fifth elution fraction (modified phage only)

**Figure 4:**
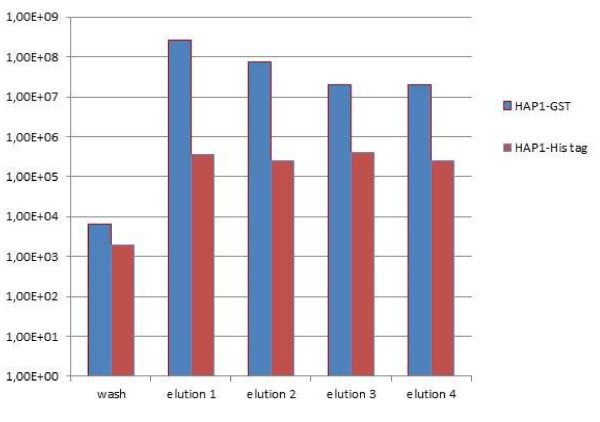
**Elution profile for the bacteriophage HAP1 modified with GST tag and purified on glutathione Sepharose compared to the same phage (HAP1) modified with a non-specific tag**. wash - phage concentration in the final washing flow-through (4^th ^litre of washing buffer) elution 1 - phage concentration in the first elution fraction elution 2 - phage concentration in the second elution fraction elution 3 - phage concentration in the third elution fraction elution 4 - phage concentration in the fourth elution fraction

**Figure 5:**
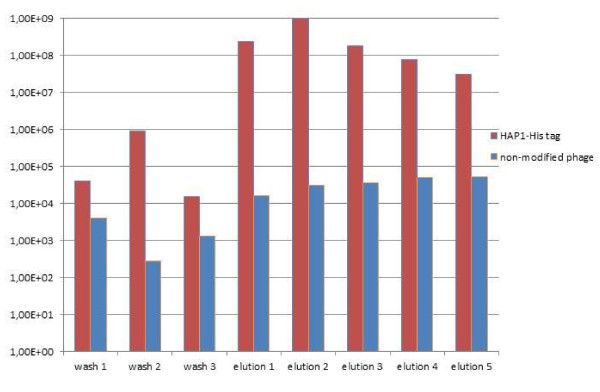
**Elution profile for the bacteriophage HAP1 modified with His tag and purified on Ni-NTA agarose compared to a non-modified phage**. wash 1 - phage concentration in the washing flow-through (1^st ^litre of washing buffer) wash 2 - phage concentration in the washing flow-through (2^nd ^litre of washing buffer) wash 3 - phage concentration in the final washing fraction (3^rd ^litre of washing buffer) elution 1 - phage concentration in the first elution fraction (imidazole 100 mM) elution 2 - phage concentration in the second elution fraction (imidazole 200 mM) elution 3 - phage concentration in the third elution fraction (imidazole 300 mM) elution 4 - phage concentration in the fourth elution fraction (imidazole 400 mM) elution 5 - phage concentration in the fifth elution fraction (imidazole 500 mM)

**Figure 6:**
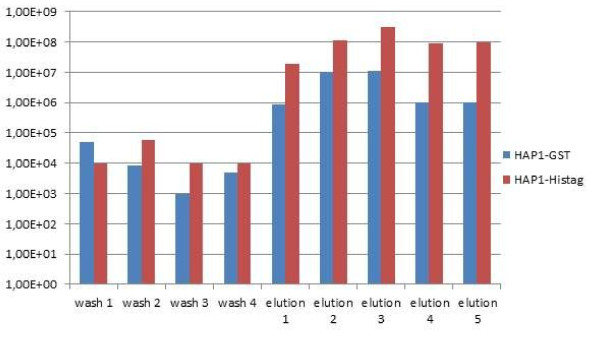
**Elution profile for the bacteriophage HAP1 modified with GST tag and purified on Ni-NTA agarose compared to the same phage (HAP1) modified with a non-specific tag**. wash 1 - phage concentration in the washing flow-through (1^st ^litre of washing buffer) wash 2 - phage concentration in the washing flow-through (2^nd ^litre of washing buffer) wash 3 - phage concentration in the washing flow-through (3^rd ^litre of washing buffer) wash 4 - phage concentration in the final washing fraction (4^th ^litre of washing buffer) elution 1 - phage concentration in the first elution fraction (imidazole 100 mM) elution 2 - phage concentration in the second elution fraction (imidazole 200 mM) elution 3 - phage concentration in the third elution fraction (imidazole 300 mM) elution 4 - phage concentration in the fourth elution fraction (imidazole 400 mM) elution 5 - phage concentration in the fifth elution fraction (imidazole 500 mM)

The affinity of modified bacteriophages to standard chromatography resins was qualified by comparing their elution profile from the specific resin with the negative controls (non-modified phages or phages modified with a non-specific tag of the same titre). Figures 3, 4, 5, and 6**present the results in the logarithmic scale**. Bacteriophage HAP1 modified with GST tag and secluded on the glutathione agarose allowed elution fractions with phage concentration more than two orders of magnitude higher than the non-modified phage (Figure 3) and even three orders of magnitude than the phage modified with a non-specific tag (Figure 4). Bacteriophage HAP1 modified with His tag and secluded on the Ni-NTA agarose (nickel resin) allowed elution fractions with phage concentration even almost five orders of magnitude higher than the non-modified phage (Figure 5) and almost two orders of magnitude higher than the phage modified with a non-specific tag (Figure 6). First-step elution fractions were tested for LPS activity; results are presented in Table 1. Approximately one order of magnitude difference between results obtained in basic conditions of washing (100 to 400 EU/ml) and prolonged washing (10 to 40 EU/ml) indicates the strict relation between washing conditions or intensity and the level of purity of obtained preparations (Table 1).

**Table 1:** Endotoxin content in bacteriophage preparations obtained in affinity chromatography of bacteriophages displaying affinity tags

Bacteriophage preparation	LPS activity (SD) [EU/ml]
Bacteriophage modified with **GST tag **and purified on **glutathione Sepharose **(Figure 3)	**186 **(124)

Bacteriophage modified with **GST tag **and purified on **glutathione Sepharose**, prolonged washing (Figure 4)	**13 **(2)

Bacteriophage modified with **His tag **and purified on **Ni-NTA **agarose (Figure 5)	**253 **(172)

Bacteriophage modified with **His tag **and purified on **Ni-NTA **agarose, prolonged washing (Figure 6)	**16 **(1)

Bacteriophage modified with **GST tag **and purified on **Ni-NTA **agarose (non-specific), prolonged washing	**40 **(2)

Bacteriophage modified with **GST tag **and purified on **glutathione Sepharose, released by proteolysis**	**363 **(47)

Bacteriophage modified with **GST tag **and purified on **glutathione Sepharose, released by proteolysis**, prolonged washing	**11 **(1)

The endotoxin assay was the Limulus Amebocyte Lysate Assay (Lonza, USA). It is based on activation of pro-enzyme in the Limulus Amebocyte Lysate by endotoxin of Gram-negative bacteria. The activated enzyme digests the non-colour substrate Ac-Ile-Glu-Ala-Arg-pNA, resulting in the molecule pNA that is spectrophotometrically detected at 405-410 nm after stopping the reaction with 25% acetic acid. The correlation between endotoxin concentration and absorbance is linear within the range 0.1-1 EU/ml; therefore all the samples were diluted to this range. All the probes were doubled for the detection. The concentration in a sample was estimated in comparison to the solutions of an endotoxin standard (supplied with the LAL kit), also doubled. Reaction conditions: 10 min. 37°C for the lysate, 8 min. 37°C for LAL substrate, then stopping the reaction and detection in 96-well plates. Only apyrogenic equipment was used (once and never reused) in the test.

The purification procedure of His tag- and GST-modified phages on Ni-NTA agarose revealed substantially higher phage concentration in elution fractions compared to final washing samples also in GST-modified (non-specific) phage. This strongly suggests a relatively high rate of non-specific phage binding (Figure 6). Therefore the first fraction of GST-modified phages after binding and washing in Ni-NTA resin was also verified for LPS activity. The purification level was comparatively good: 40 EU/ml.

Bacteriophage modified with GST was also bound to the glutathione Sepharose and released by proteolytic cleavage instead of elution. None of the phage capsid external proteins contains the short amino acid sequence recognized by the rare protease AcTEV: ENLYFQG (theoretical analysis, T4 genome sequence is available at http://www.ncbi.nlm.nih.gov/nuccore/AF158101.6). Bacteriophage was subsequently released, reaching the concentration 2 × 10^7 ^after three days and 3 × 10^8 ^after 7 days of proteolysis. This preparation was also tested for LPS activity and a favourable effect of prolonged washing was also observed (Table 1).

## Discussion

The aim of this work was to verify the possibility of applying affinity chromatography in bacteriophage purification, from the perspective of therapeutic purposes. Elution profiles of phages modified with specific affinity motifs (Figures 3,4,5 and 6) show substantially higher phage concentration in elution fractions compared to final washing samples. This indicates binding of modified phages to the affinity resins and effective elution with standard competitive agents. Thus, affinity tags can be successfully incorporated into the T4 phage capsid by the *in vivo *phage display technique and they strongly elevate bacteriophage affinity to a specific resin. Non-specific binding was also observed: unmodified phages or those modified with the non-specific tag were eluted with the titre 10^4^-10^5 ^pfu/ml. Nevertheless, the unspecific binding is 10^2^-10^5 ^times weaker than the specific one and importantly it does not interfere with the aim of preparation of purified anti-bacterial active bacteriophages for therapeutic use. In this preparation phage titres that were applied were similar to those obtained in elution fractions. The amount of the resin was generally small (as for laboratory use), but the total harvest of phages can be higher if a larger amount of resin is used (data not shown), which reflects well-known relevance in recombined protein purification procedures.

As any permanent introduction of extraneous DNA into a phage genome is strongly unfavourable for therapeutic purposes, integration of foreign motifs with the phage genome was not applied. The phage was propagated in bacteria expressing fusions of the proteins with affinity tags from bacterial plasmids, independently from the phage expression system. Nevertheless, in this work a non-essential phage gene had to be destroyed to make an easily accessible position for recombined proteins. The conditions of binding recombined Hoc with T4 ^Hoc- ^capsids were previously studied by Ren and Black [16], and by Shivachandra et al. [20,21]. The overall ratio of binding was shown to vary among 20-40 copies while there are 155 possible positions on the T4 capsid. The second group compared the frequency of phage display for N-terminal and C-terminal Hoc fusions, comparing them to mutagenesis data mapping the capsid binding site to the C-terminal domain of Hoc. They found that N-terminal fusion was about 500-fold more frequently incorporated than C-terminal and the saturation ratio was about 30:1 (250-500 nM). As the affinity of N-terminal recombined Hoc for the gp23 hexamers remains very high, it may reach the maximum number in some conditions [16,20,21]. For further simplification of the system, our group considered application of non-modified phages too. Competition between wild type proteins (expressed from the phage genome) and the recombined ones (from vectors) decreases the frequency of affinity tags' incorporation but it still offers an advantage in comparison to non-specific binding of non-modified phages (Dabrowska et al., unpublished data). Importantly, in this case there are many more target proteins (almost all capsid external ones) that should be investigated, as well as a possibility of developing the method for other T4-like phages. These issues should be proposed for further investigations and we intend to present them next, as further verification of this method's universality.

Bacteriophages were also effectively released from the glutathione Sepharose by proteolytic cleavage. The possibility of proteolytic release was designed at the stage of expression vector construction: the sequence coding for the protease-recognized motif (GAAAACCTGTATTTTCAGGGC: ENLYFQG, AcTEV protease) was introduced by a PCR primer between the *hoc *gene and the affinity motif. The proteolytic reaction "in the resin" cuts the recombinant proteins incorporated into the phage capsid, leaving the affinity motif bound to the resin and releasing the phage without the foreign motif on its capsid. This possibility is of great importance, as it allows final purified phages without artificial elements, imitating natural, non-modified ones. Potential sensitivity of a phage capsid to a rare protease cannot be excluded; therefore it should be determined previously. A theoretical analysis of T4 phage external proteins showed no sequences susceptible to cleavage (T4 genome sequence is available at http://www.ncbi.nlm.nih.gov/nuccore/AF158101.6). Additionally, in the test of phage activity (double-layer titration) after incubation with the protease no decrease of phage activity was observed (data not shown). Even such complicated bacteriophage capsids as T4 can be deprived of artificial binding motifs by specific proteolytic cleavage, released, and remain active.

Endotoxin assays show that a simple washing procedure allows most endotoxins to be removed: usual LPS contents in raw lysates exceed 10^4 ^EU/ml, and in preparation of phage purified with affinity chromatography or released by the protease or by competitive elution it is 100-1000 times lower. The intensity of washing corresponds to the decrease of endotoxin level. These results suggest that further procedure optimisation (e.g. binding and/or washing conditions) could further improve the quality of the purified product.

The phage purification problem is growing with the new interest in phage therapy that results from the crisis of antibiotic resistance in bacteria. Phages, unable to infect eukaryotic cells but strongly active against bacteria, are an alternative to antibiotic therapy of bacterial infections. They are also a prospect in cases of allergy. Available data indicate high effectiveness and safety of bacteriophage therapy. Complete independence from antibiotics' antimicrobial mechanisms was shown, i.e. bacteriophages do not follow antibiotics' cross-resistance and can be fully effective against antibiotic-resistant bacteria [23-27]. Nevertheless, one of the main limitations for phage therapy is the purification of active phages from lysates and separation from bacterial residues. Large-scale methods require simplification of procedures and the therapeutic purpose emphasizes the problem of safety. We propose affinity chromatography as an easy, efficient one-step purification strategy. The resins were adapted from standard protein affinity chromatography and are known to be effective, simple, and safe. *In vivo *phage display enables even a very large amount of phages and it reduces the preparation procedure to a simple one-step microbiological culture. Based on these initial results, affinity chromatography can be considered as a new phage purification method, appropriate for further investigations and development.

## Conclusions

Affinity tags can be successfully incorporated into the T4 phage capsid by the *in vivo *phage display technique and they strongly elevate bacteriophage affinity to a specific resin. Affinity chromatography can be considered as a new phage purification method, appropriate for further investigations and development.

## Methods

### Bacteriophages and bacteria

T4 phage from the American Type Culture Collection (ATCC, USA), HAP1 phage from the IIET Microorganisms Collection: HAP1 is a T4 phage mutant with a nonsense mutation in the *hoc *gene with no functional gpHoc. In the HAP1 *hoc *gene the transition C496→T occurs, thereby generating a nonsense mutation Gln166→ orche stop codon which was confirmed to stop incorporation of Hoc into the phage capsid [22]. *Escherichia coli *expression strains B834 and Rosetta2 (Novagen), transformed with expression plasmids carrying the *hoc *gene in N-terminal fusion with affinity tags.

### Expression vectors

Vectors were prepared using GATEWAY recombination technology following the manufacturer's instructions (Invitrogen). Cloning was carried out with polymerase chain reaction (PCR) products. Double PCR was applied for introduction of long flanking regions consisting of recombination regions and a coding region for rare protease AcTev (Invitrogen). Primers: PCR1 forward GAAAACCTGTATTTTCAGGGCAGCAGCAGCATGACTTTTACAGTTG, PCR1 reverse: GGGGACCACTTTGTACAAGAAAGCTGGGTCCTATGGATAGGTATAGATGATACC, PCR2 forward: GGGGACAAGTTTGTACAAAAAAGCAGGCTCCGAAAACCTGTATTTTCAGGGC, and PCR2 reverse: GGGGACCACTTTGTACAAGAAAGCTGGGTCCTATGGATAGGTATAGATGATACC. Entry clones were prepared with the donor vector pDONR201. Destination clones were prepared with pDEST15 (GST tag) or pDEST17 (His tag) (Figure 1). Control DNA sequencing was performed at the Institute of Biochemistry and Biophysics, Polish Academy of Sciences, DNA Sequencing and Oligonucleotide Synthesis Laboratory, Warsaw, Poland. Isolated plasmid DNA (PlasmidMini A&A Biotechnology) was applied in the reaction of sequencing (3730 DNA Analyzer, Applied Biosystems, Hitachi, DNA Sequencing KitBig Dye™ Terminator Cycle Sequencing version 1.1): 94°C for 10 s, 52°C for 20 s, 60°C for 4 min, 25 cycles; 100 ng DNA, 1 μl of 5 μM primer, 3 μl buffer, 1 μl enzyme premix, H_2_O adjusted to 10 μl (http://oligo.ibb.waw.pl).

The expression of the recombined proteins was controlled in the bacterial lysate (lysis by freeze-thawing) by SDS-PAGE of total bacterial protein profile and by test affinity chromatography with binding, washing (50 mM Na_2_HPO_4_, 300 mM NaCl, pH 7.5) and elution (glutathione buffer for GST-tag: 40 mM glutathione, 50 mM Tris, pH 8.0, or imidazole buffer for His-tag: 100-500 mM imidazole, 50 mM Na_2_HPO_4_, 300 mM NaCl, pH 7.5) followed with SDS-PAGE. Strains were used for further procedures only if effective expression of the modified proteins was noticed.

### *In vivo *phage display

Bacterial cells were grown at 37°C until OD_600 _0.7 was reached. Next they were transferred to fresh media containing 0.05-0.1 mM IPTG and approx. 10^6^-10^7 ^pfu/ml HAP1 (1:100 - 1:1000 of total culture volume), so that the induction of protein expression and phage infection took place at the same time. Infected cells were grown at 37°C for 8 hours. When bacterial cell lysis was observed, lysates were filtered and used for affinity chromatography. Control preparations: (i) not modified phage HAP1 or T4, (ii) phage HAP1 modified with a non-specific affinity tag. Control preparations had identical phage concentration compared to specific tag modified ones: **5 × 10**^**8 **^**pfu/ml**. They were purified and eluted identically.

### Purification procedure

Filtered lysates were incubated with 2 ml of glutathione Sepharose (GE Healthcare) or Ni-NTA agarose (QiaGen) overnight at 4°C. Next the unbound fraction was removed, and the resin was washed with 3 litres of sodium phosphate buffer (50 mM Na_2_HPO_4_, 300 mM NaCl, pH 7.5). Alternatively the washing procedure was prolonged: 4 litres of sodium phosphate buffer, and in the case of His-tag modification the phosphate buffer was enriched with imidazole 50 and 100 mM. Elution of specifically bound phage particles from Ni-NTA agarose was carried out competitively with a 100-500 mM gradient of imidazole. In the case of glutathione Sepharose two strategies of product release were used: (i) competitive elution with 40 mM reduced glutathione (5 eluted fractions), each incubated at least 20 min with the elution buffer, or (ii) proteolytic tag cleavage with AcTEV protease (Invitrogen) for 7 days.

Phage preparations were titrated by the two-layer method of Adams [28] and tested by Limulus amebocyte lysate assay (Lonza). Example experiments are presented in the logarithmic scale.

## Authors' contributions

AO carried out the bacteriophage phage display cultures, optimized culture conditions, carried out purification procedures, participated in phage activity determining assays, participated in data analysis and in drafting the manuscript. PB participated in optimisation of recombined proteins expression, carried out the bacteriophage phage display cultures, carried out purification procedures, participated in phage activity determining assays. AP participated in carrying out the bacteriophage phage display cultures and purification procedures, carried out phage activity determining assays. BO and AK carried out LPS analysis in purified preparations and carried out phage activity determining assays. GF participated in carrying out the bacteriophage phage display cultures and purification procedures. KD conceived of the study and designed the experiments, performed the data analysis and drafted the manuscript, afforded the expressive vectors and procedure with effective recombined proteins expression, participated in carrying out the bacteriophage phage display cultures, optimisation of culture conditions and purification procedures, participated in phage activity determining assays. All authors read and approved the final manuscript.
